# Microvessel changes after post-ischemic benign and malignant hyperemia: experimental study in rats

**DOI:** 10.1186/1471-2377-10-24

**Published:** 2010-04-16

**Authors:** Haitao Lu, Jungong Zhao, Minghua Li, Yingsheng Cheng, Yongdong Li, Xiaofang You, Yuwu Zhao

**Affiliations:** 1 Department of diagnostic and interventinal radiololgy, Shanghai sixth people's hospital, Shanghai Jiao Tong university, 600 Yi Shan Rd, Shanghai, 200233, PR China; 2 Department of neurology, Shanghai sixth people's hospital, Shanghai Jiao Tong university, 600 Yi Shan Rd, Shanghai, 200233, PR China

## Abstract

**Background:**

The present investigation was designed to elucidate the use of dynamic contrast enhanced perfusion MR imaging (DCE pMRI) in characterizing hyperemia, including microvessel changes, and to examine whether DCE pMRI can predict benign or malignant hyperemia.

**Methods:**

Sprague-Dawley rats underwent middle cerebral artery occlusion (MCAO) by intraluminal suture placement. All rats were randomized to 4 groups: MCAO for 0.5 hours followed by saline treatment (10 ml/kg; group 1); MCAO for 3 hours followed by treatment with saline (group 2) or urokinase (25000 IU/kg; group 3); and MCAO for 6 hours followed by urokinase treatment (group 4). Relative cerebral blood volume (rCBV) and relative maximum slope of increase of the signal intensity time curve (rMSI) were quantitatively analyzed from MRI. Microvessel diameter and blood-brain barrier disruption obtained by laser scanning confocal microscopy (LSCM) as well as transmission electron microscopy (TEM) were obtained for correlative study.

**Results:**

Benign hyperemia was noticed only in group 1; malignant hyperemia was seen in group 3. Although the rCBV of malignant hyperemia was slightly higher than in benign hyperemia (*P *> 0.05), the rMSI, on the other hand, was significantly lower (*P *< 0.05). Fluoro-isothiocyanate dextran (FITC-dextran) extravasations, marked glial end-foot process swelling, and significant vasodilatation were seen in malignant hyperemia, while no or mild leakage of FITC-dextran and slight glial end-foot process swelling occurred in benign hyperemia.

**Conclusion:**

Our findings indicate that DCE pMRI can characterize post-ischemic hyperemia and correlates well with microvascular damage.

## Background

Animal and human studies have demonstrated that post-ischemic hyperperfusion may occur in acute cerebral ischemia following vessel recanalization [1-7]. Sequential experimental positron emission tomography (PET) revealed three types of hyperperfusion following transient middle cerebral artery occlusion (MCAO) in cats [2]. Type 1 showed immediate hyperperfusion lasting for the whole observation period and covering a large part of the ischemic territory. In type 2, hyperperfusion was transient, and was often followed by hypoperfusion in some parts of the previously hyperperfused regions. In type 3, hyperperfused regions grew progressively(Table 1)[2]. When regions undergo transient early hyperperfusion or progressive hyperperfusion over time, they often have small infarctions, while regions with continued hyperperfusion have particularly poor outcomes [2,3]. Clinical study, on the other hand, has indicated no significant differences in the degree of clinical improvement in patients with regions of hyperperfusion versus those without [4]. The variance between experiment and clinical findings indicates that the underlying mechanism of hyperperfusion after reperfusion remains unknown. Graf. R et al. hypothesis that type 1 hyperemia was referred to manignant hyperemia, while type 2 and type 3 hyperemia were benign[2].

**Table 1:** Classification of three types of hypermia described by Graf R, et al.

Classification	Manifestation	Quality
Type 1	showed immediate hyperperfusion lasting for the whole observation period and covering a large part of the ischemic territory	malignant hyperemia
Type 2	hyperperfusion was transient, and was often followed by hypoperfusion in some parts of the previously hyperperfused regions	benign hyperemia
Type 3	hyperperfused regions grew progressively	benign hyperemia

Apart from the difference in time course and final infarction, there is no further knowledge of the differences between kinds of hyperperfusion, including microvessel changes. Furthermore, hyperperfusion data were mainly obtained by PET, there is insufficient data on the use of magnetic resonance contrast agent (MRCA)-enhanced MR imaging (MRI) with GD-DTPA to characterize hyperemia, though it is often used to evaluate BBB opening in stroke [2-5,8-10]. In addition, hyperperfusion, according to its strict definition, is an increase in cerebral blood flow (CBF). However, this definition is generally impractical, especially in human studies, because of the lack of pre-stroke CBF measurements, whereas hyperemia, defined as increase in cerebral blood volume, is more practical and often occurs during hyperperfusion [4,11]. We designed a reperfusion model with varying occlusion timesand different type of treatment to investigate the underlying mechanisms of the different kinds of hyperemia in rats following reperfusion, including their microvessel changes, to find out whether duration of ischemia or additional thrombolysis could influence the type of hyperemia, and to examine whether dynamic contrast enhanced perfusion MRI (DCE pMRI) can predict different hyperemia types [11-17].

## Methods

### Animal Model and Protocol

The experimental protocol was approved by the Institutional Animal Care and Use Committee at the Affiliated Sixth Hospital of Shanghai Jiaotong University. Focal cerebral ischemia was introduced in 33 male Sprague-Dawley rats (250 to 300 g) using an intraluminal suture occlusion model. Briefly, after the rat was anesthetized using 5 mg/kg ketamine chloride, MCAO was achieved by introducing a 4-0 paint-coated filament via the external carotid artery into the distal common carotid artery. The filament was then advanced via internal carotid artery until a faint resistance was felt (about 20 mm from the carotid bifurcation). After 0.5 hours, 3 hours, or 6 hours of occlusion, rats were treated with saline (10 ml/kg) or urokinase (25000 IU/kg, Shanghai Zhongxi Pharmaceuticals, China) intravenously over 1 hour immediately after suture withdrawal.

During the operation, rat rectal temperature was maintained at 37°C using a heating pad. All rats were randomized to 4 groups: the left middle cerebral artery (MCA) was occluded for 0.5 hours followed by saline treatment (10 ml/kg) after suture withdrawal (group 1, n = 8); the left MCA was occluded for 3 hours followed by treatment with saline (10 ml/kg; group 2, n = 8) or urokinase (25000 IU/kg; group 3, n = 8) after suture withdrawal; and the left MCA was occluded for 6 hours followed by urokinase treatment (25000 IU/kg; group 4, n = 9). MRI was performed 10 minutes before reperfusion and both 3 hours and 10 days following vessel reperfusion.

### MRI

MRI was performed using a GE Signa 1.5 T system with a 3-inch surface coil. T1- and T2-weighted images were obtained using a 3-mm thick section and 0.5 mm intersection space with a 256 × 160 matrix. Diffusion-weighted images (DWI) were acquired with 8000 ms repetition time, 97.8 ms echo time, and b value of 1000 s/mm^2^. DCE pMRI was obtained by a gradient-echo planar sequence during the first pass of a standard bolus of gadopentetate dimeglumine (Gd-DTPA), 0.2 mmol/kg (about 0.5 mL), which was injected through the femoral vein after the first 5 baseline scans. Five images were acquired with 50 repetitions, which resulted in a total acquisition time of 56 seconds. Repetition time was 1000 ms and echo time was 34.7 ms. To avoid image deformity and improve the signal-to-noise ratio, both DWI and DCE pMRI were acquired with a matrix 64 × 64, field of view (FOV) 5 × 5, thickness 3 mm, and intersection space 0.5 mm. Post-contrast axial T1-weighted images were acquired following the acquisition of DCE pMRI data.

To quantitatively analyze DCE pMRI, regions of interest (ROIs; 2 mm^2^) were hand-drawn to cover the parietal cortex and striatum, both of which are usually affected in the MCAO model, as well as homologous areas on the contralateral side. A signal intensity time curve was obtained over each ROI. The cerebral blood volume and maximum slope of increase of the signal intensity time curve (which indicates the velocity of signal intensity return to baseline) were calculated automatically at the workstation (SUN Microsoft Ultra 60) using commercially available post-processing software (Functool 2.5.36d, GE Medical Systems). The relative cerebral blood volume (rCBV) and the relative maximum slope of increase (rMSI) were produced using the following formulas: rCBV = CBV on the ipsilateral side/CBV on the contralateral side; and rMSI = MSI on the ipsilateral side/MSI on the contralateral side. An rCBV increase of more than 20% compared with the value on the contralateral normal side was regarded as hyperemia, as described elsewhere, and the results were divided into 5 types according to the appearance of perfusion changes: normal perfusion (N), early hyperemia, late normal perfusion (I), early normal perfusion, late hyperemia (II), persistent hyperemia (III), and persistent hypoperfusion (IV) [5].

### Light Microscopy and Transmission Electron Microscopy (TEM) Studies

Two animals randomly selected from each group were killed after 3 hours and 10 days of recanalization. The tissues where the rCBV and rMSI had been monitored were block-dissected, fixed brain tissues were serially sectioned axially at 500 μm, and then were rinsed, osmicated, dehydrated, and embedded in Epon-Araldite plastic (Shanghai Colophony Company, Shanghai, China). Ultra-thin sections of 90 nm were then cut from the same blocks and stained with uranyl acetate and lead citrate for transmission electron microscopy (TEM) analysis.

Our targets were brain capillaries, so ultra-thin sections were first examined at low power to find capillaries with walls sectioned at 90° to the long axis of their lumina. Photomontages of brain capillaries were then made and each animal had 2 sections with 10 photomontages. Morphometric analysis was performed using the LEICA Qwin imaging analysis system (Germany). Measurements were made of luminal surface area (LSA) and pericapillary glial sheath surface area (PGSA) as described elsewhere [7].

The remaining parts of the fixed brain tissue were sectioned at 5 μm and stained with hematoxylin and eosin for light microscopy. Volume of cerebral infarction was measured using the LEICA Qwin imaging analysis system as well. Each H& E stained section was evaluated at 10 × magnification, the area of infarction were calculated on H&E stained section by tracing the area on computer screen, and the volume were determined by integrating the appropriate area with the section interval thickness.

### Laser Scanning Confocal Microscopy (LSCM) Examination

Ten days following vessel reperfusion, fluoro-isothiocyanate dextran (FITC-dextran, SIGMA, USA; 1 ml of 50 mg/ml preparation) was administered intravenously to 2 animals in each group. After FITC-dextran circulated for 1 minute, the brains were rapidly removed and placed in 4% paraformaldehyde at 4°C for 24 hours. Axial sections (50 μm thick) were cut on a vibration microtome, and the sections corresponding to MRI were analyzed by laser scanning confocal microscopy (LSCM; LSM510-Zeiss, Germany). The green fluorochromes in each section were excited by a laser beam at 488 nm.

Using tissue samples from each rat, 10 sections at 1 mm intervals were screened under 5× objective lens to find the areas corresponding to where rCBV and rMSI had been monitored. Under 10 × magnifications, areas of interest in the infarct region as well as the homologous areas on the contralateral side were scanned with a 1024 × 1024 matrix in the x-y direction, along the z-axis with a 1 μm step size. After scanning, three-dimensional reconstructions were employed to produce microvascular architecture that was labeled by FITC-dextran. The diameters of microvessels in the infarct zone as well as in homologous contralateral areas were calculated.

### Statistical Analysis

A statistical package (SPSS, version 13.0 for windows; SPSS, Ill, USA) was used for statistical analysis. Comparisons of measured rCBV, rMSI, and diameter of microvessels between different groups were performed by one-way ANOVA or paired-samples t-test. Values are given as mean ± SD, and *P *< 0.05 was accepted as significant.

## Results

**Figure 1:**
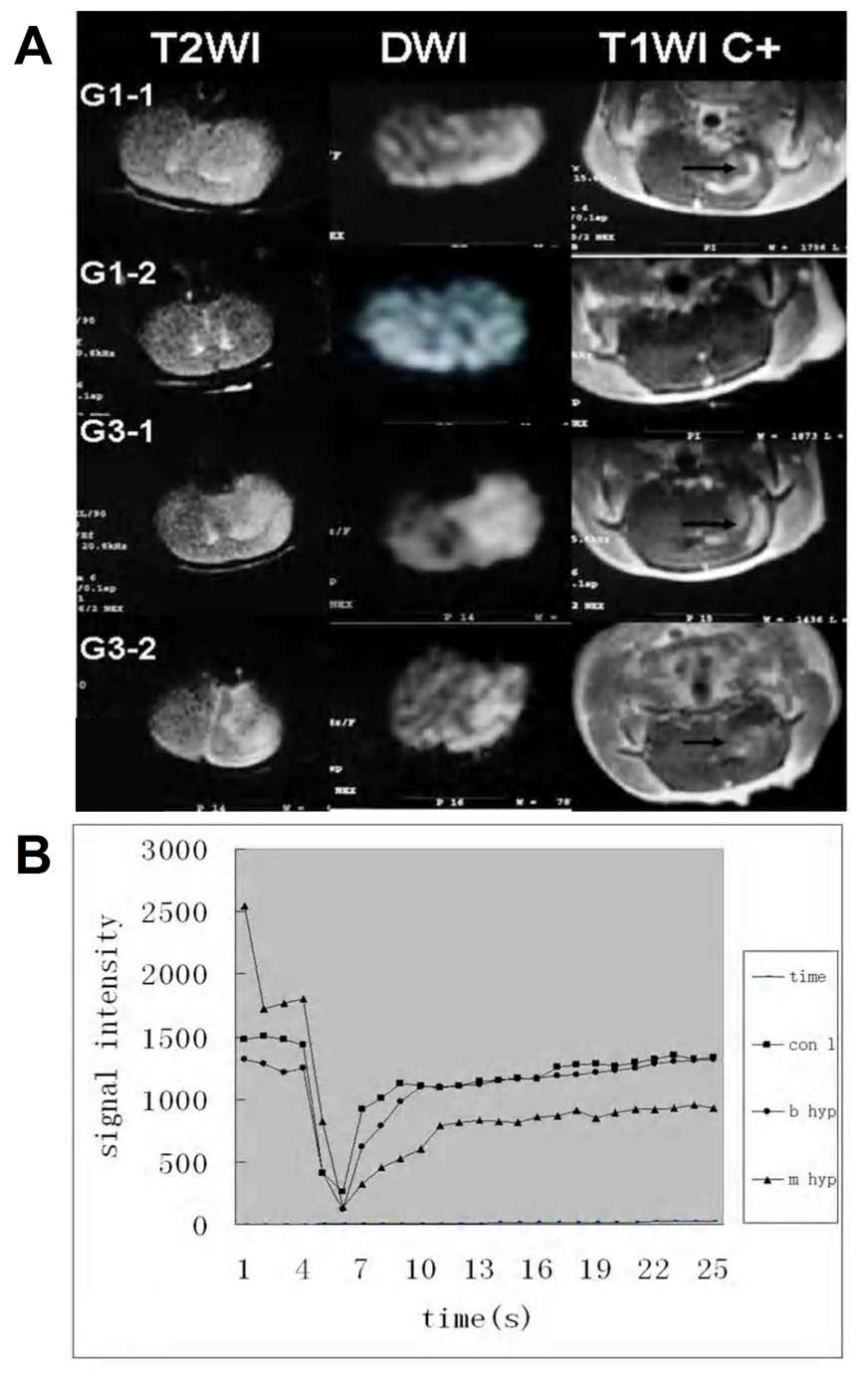
**MR images and signal-intensity time curves in different types of hyperemia**. A, Slight hyperintensity of the left hemisphere was found on T2-weighted imaging and DWI, respectively, with remarkable enhancement on contrast-enhanced T1-weighted imaging (arrow head) in G1-1 and G3-1. Ten days after reperfusion, signal intensity returned to normal on T2-weighted imaging and DWI, and no enhancement was found on contrast-enhanced T1-weighted imaging in G1-2. However, the hyperintensity remained on T2-weighted imaging and DWI, with continuous enhancement on contrast-enhanced T1-weighted imaging in G3-2 (arrow head). B, Signal intensity plotted against time for the infarct region (dots in G1-1, triangles in G3-2) and homologous areas on the contralateral side (squares). The lines represent the curves derived by using the DCE pMRI. The return to baseline is lower in G3-2 than in G1-1, suggesting high permeability of the BBB. G1-1 = three hours after reperfusion of rats in group 1; G1-2 = ten days after reperfusion of rats in group 1; G3-1 = three hours after reperfusion of rats in group 3; G3-2 = ten days after reperfusion of rats in group 3; T1WI C+ = contrast-enhanced T1-weighted imaging; Con l = contralateral side; b hyp = benign hyperemia; m hyp = malignant hyperemia.

Two rats in group 3 and three rats in group 4 died owing to large infarctions before the end of the experiment, these 5 rats were eliminated from the study. Ten days after reperfusion, the initial hyperintense areas on DWI returned to normal in 3 of 8 rats in group 1, and remained hyperintense in the other groups. In both parietal cortex and striatum, rCBV reduced significantly in groups 2, 3, and 4 before reperfusion compared with that in group 1, and only mild reduction of rCBV was noticed in group 1 before reperfusion compared to the unaffected hemisphere. After reperfusion, hyperemia was visualized only in groups 1 and 3, while hypo- or normal perfusion was found in the other groups (Figure 1A, Table 2). Type I hyperemia was noticed in the infarct region in group 1, and type II and III were seen in the infarct region in group 3. Although the degree of rCBV in group 3 was slightly higher than that in group 1, no significant difference was found between them (*P *> 0.05). The rMSI, on the other hand, was significantly lower in group 3 compared with that in group 1 (*P *< 0.05) (Figure 1B). Mean infarct volumes was 20 mm^3 ^± 11 in group 1, 118 mm^3 ^± 32 in group 2, 132 mm^3 ^± 49 in group 3 and 287 mm^3 ^± 71 in group 4, no hemorrhagic transformation was found in any group.

**Table 2:** Dynamic contrast enhanced perfusion MRI changes before and after reperfusion

Group	n	rCBV(%)	rMSI(%)
		
		mean ± SD	mean ± SD
**Group 1**			
Before	8	86.0 ± 8.2*	110.6 ± 12.4
3 h	8	141.5 ± 22.3	80.7 ± 19.1
10 d	8	102.8 ± 11.3	109.0 ± 6.2**
**Group 2**			
Before	14	46.2 ± 16.9*	69.7 ± 30.1
3 h	8	88.3 ± 13.8	74.8 ± 19.9
10 d	8	78.3 ± 9.3	75.0 ± 19.5
**Group 3**			
Before	14	46.2 ± 16.9*	69.7 ± 30.1
3 h	6	127.8 ± 13.6	82.5 ± 30.7
10 d	6	173.5 ± 45.2	51.8 ± 10.9**
**Group 4**			
Before	6	23.6 ± 16.8*	59.4 ± 27.5
3 h	6	103.4 ± 26.6	64.1 ± 28.9
10 d	6	80.0 ± 28.6	76.0 ± 24.3

rCBV = relative cerebral blood volume; rMSI = relative maximum slope of increase; before = before reperfusion; 3 h = three hours after reperfusion; 10 d = ten days after reperfusion.

*Significant difference (*P *< 0.5) among the different groups before reperfusion.

**Significant difference (*P *< 0.5) between group 1 and group 3 ten days after reperfusion.

**Figure 2:**
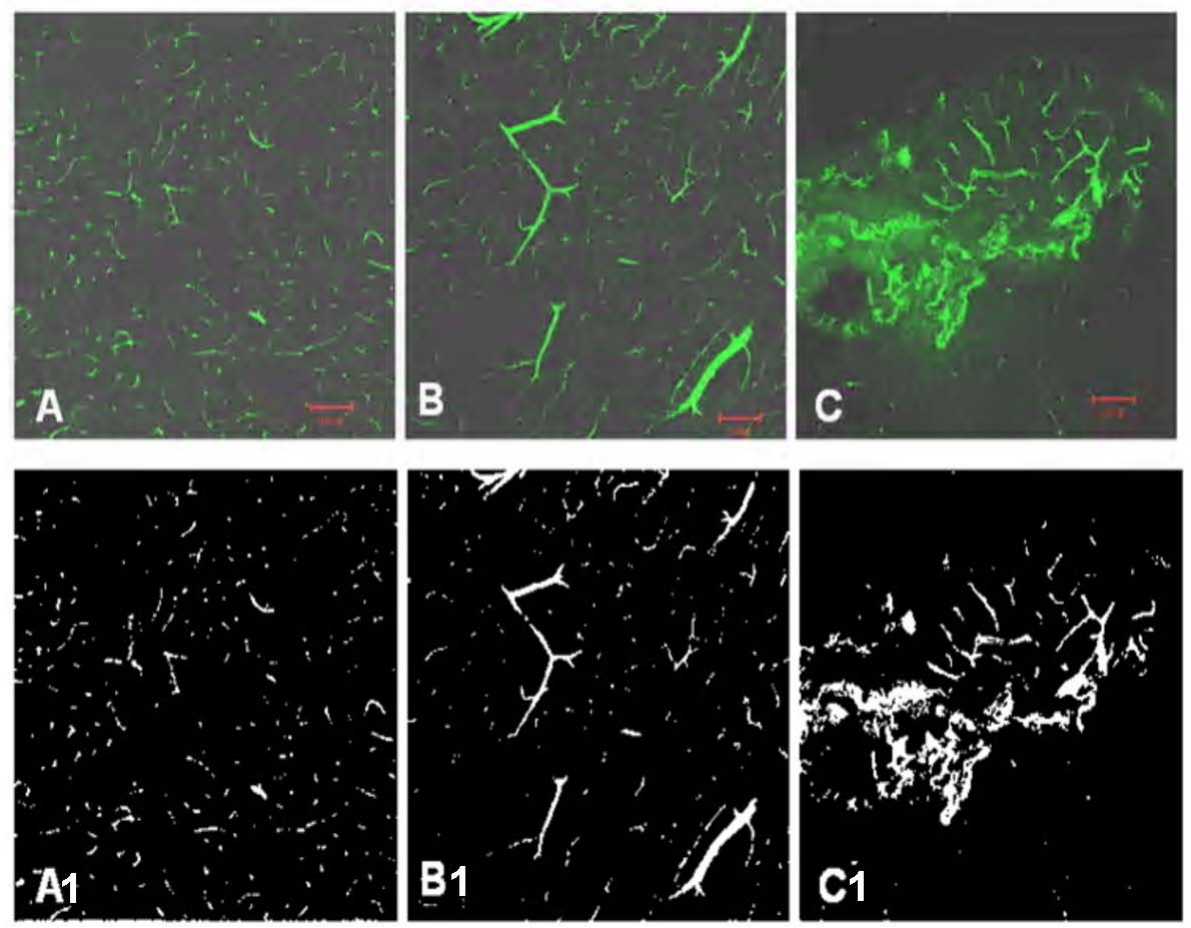
**Cerebral microvessel changes 10 days after reperfusion**. A, Cerebral microvessel perfusion obtained by FITC-dextran was compared with normal cerebral microvessels in homologous contralateral areas. B, No leakage of FITC-dextran and slight dilation of microvessels occurred in benign hyperemia. C, The area showing hyperemia on DCE pMRI demonstrates FITC-dextran extravasations as well as significant vasodilatation in group 3. Bar = 100 μm.

According to LSCM, all areas showing type II and type III hyperemia demonstrated FITC-dextran extravasations as well as significant vasodilatation, while no or mild leakage of FITC-dextran and slight dilation of microvessels occurred in type I hyperemia (P < 0.05; Figure 2, Table 3).

**Table 3:** Diameter of microvessels in different groups according to LSCM.

Group	diameter (μm) (mean ± SD)
Group 1	16.2 ± 3.5*
Group 2	17.1 ± 2.7
Group 3	46.0 ± 22.4*
Group 4	24.4 ± 6.8
Normal	6.7 ± 1.5

Two animals with 20 sections in each group were calculated.

Normal = the homologous areas on the contralateral side in all four groups.

* Significant difference (*P *< 0.5) between group 1 and group 3 ten days after reperfusion.

**Figure 3:**
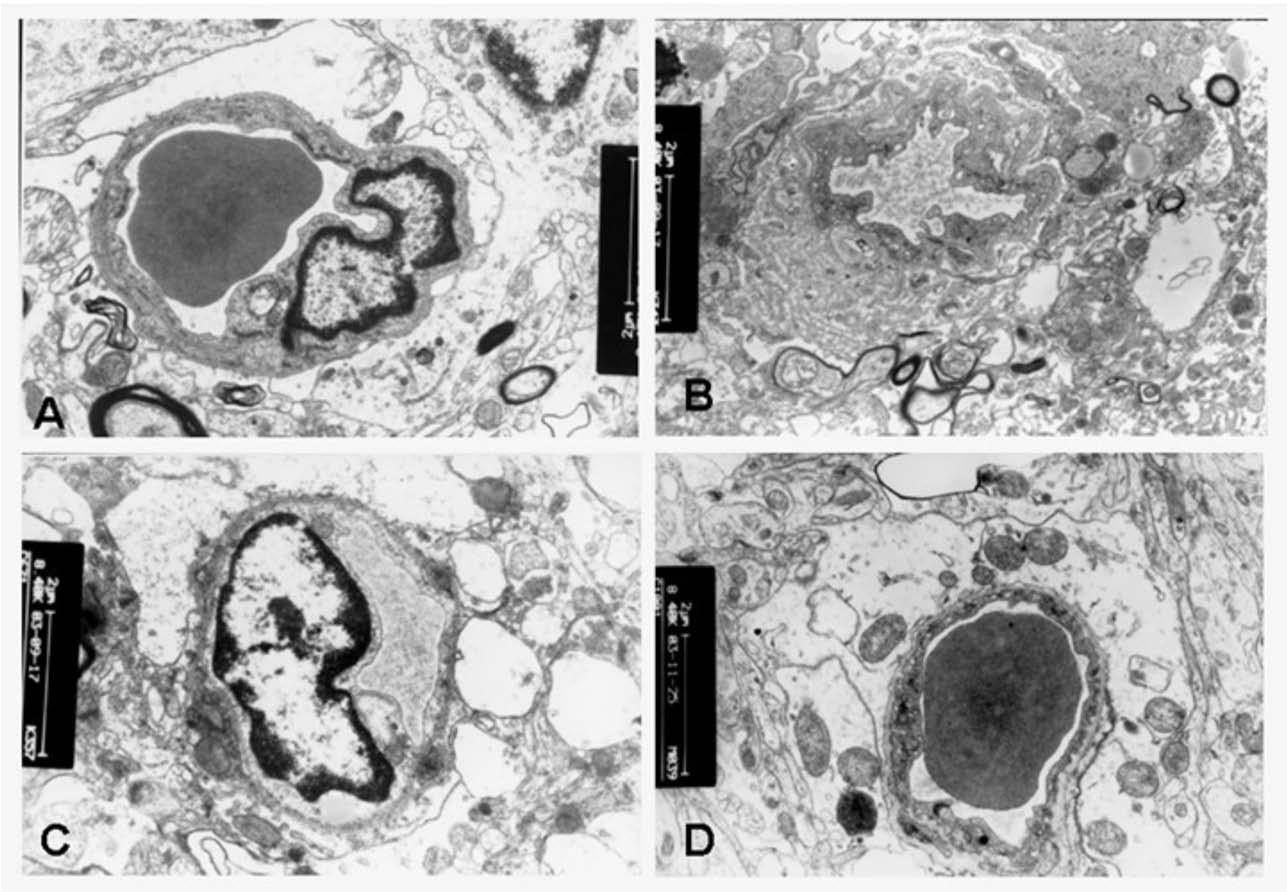
**Ultrastructural alterations after reperfusion**. A, Three hours after reperfusion, slightly swollen glial end-foot processes were observed and capillaries showed opening lumen in group 1. B, Ten days after reperfusion, swelling of glial end-foot processes attenuated and an irregular luminal surface was observed in the same group. C, In group 3, enlargement of endothelial cell nuclei as well as marked edema of glial end-foot processes was found. D, Ten days after reperfusion, the edema became more severe in group 3. Bar = 2 μm.

Three hours after reperfusion, group 1 showed mild astroglial end-foot process edema, and capillaries had an irregular luminal surface. Ten days after reperfusion, the end-foot process edema decreased slightly, and LSA decreased from 6.4 μm^2 ^to 2.1 μm^2 ^with no change in PGSA (Table 4). In group 3, severe astroglial end-foot process edema was found even 10 days after reperfusion, and LSA decreased from 17.4 μm^2 ^to 6.6 μm^2^; furthermore, PGSA increased steadily (Figures 3). According to pathology and changes in infarct volume, type I hyperemia was referred to benign hyperemia, while type type II and III hyperemia were malignant.

**Table 4:** Morphological data of the infarct region in different groups by TEM.

Group	LSA (μm^2^) (mean)	PGSA (μm^2^) (mean)
**Group 1**		
3 h	6.4	10.3
10 d	2.1	10.4
**Group 2**		
3 h	4.3	10.9
10 d	3.1	9.7
**Group 3**		
3 h	17.4	11.5
10 d	6.6	14.9
**Group 4**		
3 h	4.2	11.8
10 d	0.7	11.4

LSA = luminal surface area; PGSA = pericapillary glial sheath surface area; 3 h = three hours after reperfusion; 10 d = ten days after reperfusion. At different time points in each group, n = 2.

## Discussion

Our study provides unique insight into post-ischemic hyperemia. We found that post-ischemic hyperemia mainly occurred in group 1 and group 3, while hypo- or normal perfusion was found in the other groups. Furthermore, benign hyperemia was noticed in rats after 0.5 hours of MCAO following recanalization, but malignant hyperemia occurred after 3 hours of MCAO with additional thrombolysis. These results indicate that longer times of ischemia with additional thrombolysis may be associated with malignant hyperemia, and hyperemia does not develop after prolonged ischemia because of a greater tendency for lack of reflow.

Our data suggest that the different kinds of post-ischemic reperfusion resulted from different microvessel conditions. Benign hyperemia (i.e. reactive hyperemia) mainly occurred after transient ischemia and might be caused by neurogenic-mediated vessel dilatation [18]. The cerebral resistance vessel (e.g. arteriole) was unable to constrict during reperfusion, but the integrity of microvessels remained, and when the neurogenic reaction ceased, the microvessels recovered their normal condition, so no hyperemia was found in the later stage. After a longer MCAO, polymorph nuclear leukocytes adhered and fibrin formations and platelet-fibrin aggregated, decreasing focal reflow in both capillaries and postcapillary venules after suture withdrawal from MCA, although the microvessels dilated [1]. This would explain why there was no hyperemia in group 2. After thrombolytic treatment, microvessels were fully recanalized, which caused the initial hyperemia in group 3, but the release of vasodilating substances might contribute to persistent vessel dilatation and the later stage of hyperemia in group 3 [19]. However, three hours after MCAO, the primary microvessel permeability barrier was lost, and swollen endothelial cells and astrocytes prevented microvessel recovery. Persistent and prolonged hyperemia could cause additional damage by inducing edema via leaking vascular endothelium, resulting in malignant hyperemia. After much longer ischemia (i.e. 6-hour MCAO), extrinsic compression from edema and endothelial cell swelling compressed the lumen of the microvessels, inhibiting hyperemia; this may be why no hyperemia was found in group 4.

Post-ischemic hyperemia, defined as more than a 20% increase in CBV compared with the normal contralateral side by MRI, has been documented in one previous study in cats [5]. Although a different kind of hyperemia was found in that study, all animals only underwent one hour of MCAO. In our study, rats undergoing varying occlusion times were evaluated following suture withdrawal with or without thrombolysis, and all three type of hyperemia were found, but early hyperemia, late normal perfusion, and persistent hyperemia caused different outcomes, which differs from the previous study [5]. This might owe to the longer time of ischemia and longer interval of MRI than that in the previous study.

Our results also indicated that different types of hyperemia had different outcomes. Reperfusion after 0.5 hours of MCAO, resulting in a brief hyperemia period and a fast normalization of flow, did not cause gross infarction, or the final infarct was slight. During longer periods of MCAO, persistent or late hyperemia was observed, with an increase in infarct volume and severely swollen glial end-foot processes and remarkable leakage of FITC-dextran. Forced reperfusion by reopening the MCA could not salvage already irreversibly damaged tissue, but caused additional damage, and this effect was aggravated in paralyzed vessels. Suppression of post-ischemic malignant hyperemia might significantly reduce blood-brain barrier (BBB) disruption and ischemic edema [1]. In addition, due to release of vasoactive neuropeptides from perivascular nerves, blockage of perivascular sensory nerve fibers or their constituent neuropeptides might attenuate hyperemia and cerebral edema as well [18]. Previous experimental and clinical studies have demonstrated post-ischemic hyperemia using DCE pMRI, and their data focused on the evaluation of rCBV [4,5]. In our study, rCBV was higher in malignant hyperemia than in benign hyperemia, with no significant difference, while there was a significant difference in rMSI between the two types. In comparison with LSCM, the decrease in rMSI coincided with FITC-dextran leakage. DCE pMRI can monitor the first pass of contrast agent (Gd-DTPA) through target tissue: a more substantial initial signal intensity drop indicates higher rCBV, and the delayed return to baseline often suggests high permeability of brain tumors [20,21]. Although the volume transfer constant (*K*^trans^) is a good method to measure the permeability of BBB, our results suggest that rMSI, indicating the velocity of the signal intensity time curve return to baseline, is a simple index related to BBB permeability, and might be used as an adjunctive parameter to differentiate benign and malignant hyperemia even if no significant rCBV difference is evident at a particular time point [21].

Our results also suggest that when patients undergo a longer time of ischemia, complete recanalization may result in malignant hyperemia. When this kind of hyperemia is demonstrated by DCE pMRI after thrombolysis, anti-reperfusion injury measurements should be taken to reduce exacerbation of ischemic edema caused by post-ischemic hyperemia.

Our study has some limitations. No sham operation served as a control and quantitative DCE pMRI measurements are not available at our hospital. Therefore, we used only relative perfusion parameters. Although rCBV and rMSI do not perfectly represent cerebral blood flow and the degree of BBB disruption, many hospitals use them because they are easily calculated using commercial software. An additional limitation of our study is that we monitored the CBV of rats after reperfusion only at two time points. Serial imaging studies performed at more time points will be required to further characterize the time course. What's more, the results for LSCM and TEM are based on only 2 animals of each group.

## Conclusion

Our study indicates that benign and malignant post-ischemic hyperemia, having different pathogeneses and different outcomes, could be identified using DCE pMRI, especially when rMSI decreased remarkably. This indicated the BBB disruption, and special management should be taken to prevent cerebral edema in such cases. These findings provide a novel measurement which can be used to characterize post-ischemic hyperemia and which correlates with microvascular damage.

## Competing interests

The authors declare that they have no competing interests.

## Authors' contributions

HL: participated in the design of the study and performed the statistical analysis. JZ: participated in the design of the study and performed the statistical analysis. ML: conceived of the study, and participated in its design and coordination. YC: conceived of the study, and participated in its design and coordination. YL: conceived of the study, and participated in its design and coordination. XY: conceived of the study, and participated in its design and coordination. YZ: participated in the design of the study and performed the statistical analysis. We declare that all authors have read and approved the final manuscript.

## Pre-publication history

The pre-publication history for this paper can be accessed here:

http://www.biomedcentral.com/1471-2377/10/24/prepub
